# Garbage in, Garbage Out: Data Collection, Quality Assessment and Reporting Standards for Social Media Data Use in Health Research, Infodemiology and Digital Disease Detection

**DOI:** 10.2196/jmir.4738

**Published:** 2016-02-26

**Authors:** Yoonsang Kim, Jidong Huang, Sherry Emery

**Affiliations:** ^1^ Health Media Collaboratory Institute for Health Research and Policy University of Illinois at Chicago Chicago, IL United States

**Keywords:** social media, precision and recall, sensitivity and specificity, search filter, Twitter, standard reporting, infodemiology, infoveillance, digital disease detection

## Abstract

**Background:**

Social media have transformed the communications landscape. People increasingly obtain news and health information online and via social media. Social media platforms also serve as novel sources of rich observational data for health research (including infodemiology, infoveillance, and digital disease detection detection). While the number of studies using social data is growing rapidly, very few of these studies transparently outline their methods for collecting, filtering, and reporting those data. Keywords and search filters applied to social data form the lens through which researchers may observe what and how people communicate about a given topic. Without a properly focused lens, research conclusions may be biased or misleading. Standards of reporting data sources and quality are needed so that data scientists and consumers of social media research can evaluate and compare methods and findings across studies.

**Objective:**

We aimed to develop and apply a framework of social media data collection and quality assessment and to propose a reporting standard, which researchers and reviewers may use to evaluate and compare the quality of social data across studies.

**Methods:**

We propose a conceptual framework consisting of three major steps in collecting social media data: develop, apply, and validate search filters. This framework is based on two criteria: retrieval precision (how much of retrieved data is relevant) and retrieval recall (how much of the relevant data is retrieved). We then discuss two conditions that estimation of retrieval precision and recall rely on—accurate human coding and full data collection—and how to calculate these statistics in cases that deviate from the two ideal conditions. We then apply the framework on a real-world example using approximately 4 million tobacco-related tweets collected from the Twitter firehose.

**Results:**

We developed and applied a search filter to retrieve e-cigarette–related tweets from the archive based on three keyword categories: devices, brands, and behavior. The search filter retrieved 82,205 e-cigarette–related tweets from the archive and was validated. Retrieval precision was calculated above 95% in all cases. Retrieval recall was 86% assuming ideal conditions (no human coding errors and full data collection), 75% when unretrieved messages could not be archived, 86% assuming no false negative errors by coders, and 93% allowing both false negative and false positive errors by human coders.

**Conclusions:**

This paper sets forth a conceptual framework for the filtering and quality evaluation of social data that addresses several common challenges and moves toward establishing a standard of reporting social data. Researchers should clearly delineate data sources, how data were accessed and collected, and the search filter building process and how retrieval precision and recall were calculated. The proposed framework can be adapted to other public social media platforms.

## Introduction

Social media have transformed public and interpersonal communications. The Internet and social media have quickly become major sources of health information [1-3], providing both broad and targeted exposure to such information as well as facilitating information-seeking and sharing. As people increasingly turn to social media for news and information [4,5], these platforms can serve as novel sources of observational data for infodemiology, public health surveillance (infoveillance, digital disease detection) [6-11], tracking health attitudes and behavioral intention [6, 7, 9,12-16], and measuring community-level psychological characteristics related to health outcomes [17,18].

While Facebook remains the most commonly used social media platform, varying privacy settings and complex application programming interface (API) streams make the collection and interpretation of Facebook data for observational research extremely challenging. In contrast, Twitter, which is by nature a much more public-facing platform, has millions of active users who provide rich qualitative data in the content of microblog messages (tweets) as well as important quantitative data embedded in the metadata. Metadata fields describe the reach and patterns of the diffusion of a given message, along with some limited characteristics of the users posting messages. Similarly, YouTube has millions of active users who view, post, rate, and comment on its rich video content and advertising. A simple search of any social media platform can provide a tantalizing bounty of information. Yet despite the rich potential of these platforms for research and analysis, methods for collecting, cleaning, and reporting social media data can vary widely, making the evaluation and comparison of studies using those data difficult at best.

Social media data collection in infodemiology is usually defined by the keywords and search filters used to retrieve data from the platform [6]. As such, search filters are the lens through which we can observe what and how people communicate. If our lens is appropriately focused, we can identify content of interest and avoid collecting a lot of irrelevant information. Conversely, if our search is too narrow, we may miss important data and our conclusions may be biased. If it is too broad, we risk collecting a lot of irrelevant and potentially misleading material.

A search filter is a set of keywords integrated with search rules that specify search strategies. While there is an intuitive simplicity in identifying keywords and search rules for a given research question, that seeming simplicity is deceptive. First, keyword selection is not simple. Language and popular culture vary by age, socioeconomic status, race/ethnicity, geographic location, etc. The language used on social media is often colloquial, creative, and varying. Further, users communicate differently across platforms, partly driven by the norms and technical constraints unique to each platform, and partly driven by the social function of each platform [19]. For example, Twitter users are limited to 140 characters and typically post short messages using abbreviations and slang terms. Facebook posts can be longer and thus are more likely to contain multiple, different words for a single construct. YouTube videos are posted with titles and often tagged by the poster with keywords. Instagram posts typically have multiple hashtags that offer some indication of the content. If a researcher is not fluent—or at least familiar—with the language norms of a particular platform, their search filter may be overly broad, too narrow, or simply off-topic.

The keyword is only one part of a filter; without practical rules, an intuitive search term can retrieve a lot of irrelevant information. For example, in tobacco research, the term “smoking” is critically important to any search for relevant content. But without further rules to refine that term, the keyword will retrieve plenty of content about “smoking marijuana,” “smoking ribs,” and “smoking hot girls” [9,12]. A sentiment analysis of data retrieved with the broad “smoking” term would produce different results from data retrieved with a search filter that excluded other key terms that appear in close proximity to “smoking.” Therefore, developing reliable search filters requires a rigorous process to weed out irrelevant content and assure high-quality data collection [20].

While many studies have reported lists of keywords used to retrieve social data [7-10,12-16,21-24], few describe development of search filters [7,9,15,22,23], and fewer yet attempt assessment of search filters by providing what fraction of collected data are relevant [9,15,16,22,23]. One study provided the probabilities of losing possible relevant tweets by removing certain keywords [22] but did not fully assess their search filter.

Because the quality of social data and the interpretation of subsequent analyses depend on the quality of search filters, it is imperative for social media researchers to provide evidence of the quality and scope of their data: face validity is not sufficient. Computer scientists, communication researchers, and librarians, among others, use precision and recall as measures of search filter quality [20,25,26]. Most studies that use social media data, however, do not attempt to objectively assess the quality of their data. There is often confusion about the meaning of precision and recall because they are used to assess the performance of machine learning classifiers or disease screening tests, which is different from what we aim to assess: the quality of retrieved data. To avoid confusion, we define the precision and recall used to access the quality of retrieved data as the *retrieval precision* and *retrieval recall*. We use the terms precision/recall and retrieval precision/recall interchangeably throughout the paper unless clear distinction is needed.

In studies that do assess validity, search filters are compared against a gold standard that is typically human coding. No studies so far have considered the fact that human coders can make errors. Some errors associated with coding social media contents are inevitable despite well-trained human coders. An imperfect gold standard may cause bias in the validity assessment [27]. While a perfect coding standard may be impractical, it is important that researchers are transparent and consistent about how they report the quality of coding and the strengths and limitations of their benchmark.

In this paper, we describe a framework for the collection and assessment of social media data. The goal is to move toward a reporting standard that researchers and reviewers can use to compare the quality of data retrieved and analyzed across different studies. For illustrative purposes, we use data collection from Twitter to illustrate concepts that can be adapted for other text-based social media platforms open to public. Further, we use electronic cigarette (e-cigarette) content as a working example of a salient public health topic that is rapidly changing, with constantly emerging new brands and new slang [9,12] that challenge researchers’ grasp of the language that social media users use to communicate about and market these products.

Below, we first propose a conceptual framework for social media data collection. Within this framework we describe the development of search filters, illustrate the calculation of retrieval precision and recall, and illustrate common challenges and potential workarounds. Next, we apply our framework to a real-world example using data on e-cigarette content: approximately 4 million tweets retrieved from the Twitter firehose. Finally, we discuss the challenges of applying this rigorous approach to data collection and quality assessment and propose a checklist for reporting data preparation.

## Methods

### Conceptual Framework for Social Data Collection and Quality Assessment

We propose a framework that consists of three major steps to develop and validate search filters (see Table 1). The proposed framework is designed for users who can access partial or full data streams and can be applied to a human-based process that mainly relies on human judgment and coding, and an automated process supported by machine learning techniques and less human judgment [28].

**Table 1:** A framework for Twitter data collection and validation.

Step	Details
Develop search filter	1. Build a list of search keywords: (a) Generate a list of candidate keywords based on expert knowledge, systematic search of topic-related language, and other resources, (b) Screen the keywords by examining relevance and frequency of posts, (c) Discard keywords that return posts with high proportion of irrelevant contents or relatively low frequency, and (d) Add and screen new keywords when new relevant terms and phrases emerge.
	2. Integrate keywords with search rules (eg, Boolean operators) for a more focused search.
Apply search filter	3. The search filter retrieves and splits data into a retrieved set and an unretrieved set.
Assess search filter	4. Cross-tabulate data by gold standard and search filter: (a) Randomly sample from retrieved and unretrieved data; stratified sampling may be applied, (b) Manually code sampled data to determine relevance in both of retrieved and unretrieved sets, (c) Cross-tabulate sampled data by human-coded relevance (coded relevant vs irrelevant) and search filter retrieval status (retrieved vs unretrieved).
	5. Compute retrieval precision and retrieval recall.

### Develop Search Filter

#### Build a List of Keywords

The first step in developing search filters is keyword selection. Depending on the research topic, keywords should be generated based on expert knowledge and systematic search of topic-related language. It is helpful to brainstorm and categorize keywords into subgroups. In our e-cigarette example, we categorized e-cigarette–related keywords into three subgroups: devices, brands, and behaviors.

Keyword selection also depends on social media platforms from which data are gathered. Twitter data raise unique challenges in keyword selection due to the limited number of characters allowed in a message. Twitter users often shorten messages they post by using hashtags, abbreviations, colloquialisms, and slang terms. For example, the term “square” is slang for cigarettes. A researcher without prior knowledge of this term might create a search filter that does not include the term, likely missing out on many tobacco smoking-related contents. It is therefore crucial for researchers to keep up with current abbreviations, colloquial expressions, and slang terms in their research topics. Resources such as urban dictionary [29] and a diverse team of researchers are essential to generate and understand such keywords.

Despite these efforts, many important terms may still be left out. It is therefore necessary to strategically employ broad search terms rather than highly specific terms/expressions. For example, a tweet like “A girl sitting next to me smokes squares” will be captured using a broad term “smoke” even if one does not know the term “square.” Although using broad search terms like “smoke” generates many irrelevant tweets, it reduces the probability of omitting relevant content. This is particularly useful when researchers do not have access to historical archives of social media platforms and are collecting data via streaming.

The list of keywords should be further screened and updated iteratively based on relevance and frequency. The keywords that return relatively few tweets (eg, <10 over a month) or that return a small proportion of relevant tweets (eg, <30% precision) may be discarded. That is, the signal (relevant data) to noise (irrelevant data) ratio should be considered [22] and proper thresholds may depend on research questions. New keywords should be added to the list when new relevant terms and phrases emerge (eg, new e-cigarette brands, frequent co-occurring terms). Repeating Steps 1-4 of *Build a list of search keywords* in Table 1 improves the quality of keywords and provides a good understanding of how social media users talk about a specific topic. If the data are collected for surveillance or forecasting, keywords should be updated periodically and related media coverage (if any) should be accounted.

#### Integrate Keywords With Search Rules

A search filter is a combination of keywords and search rules. Integrating keywords with search rules greatly improves the ability of search filters to retrieve relevant messages. Search rules can be used to weed out irrelevant messages retrieved by broad terms. For example, in tobacco research, irrelevant tweets can be excluded by specifying that terms such as “barbeque” or “marijuana” do not appear in the tweets, while relevant tweets could be kept if a tweet contains both terms “smoke” and “square.” These search rules can be constructed using the Boolean operators (AND, OR, NOT) and data pre-processing techniques such as n-grams or proximity operator.

### Apply Search Filter

**Figure 1:**
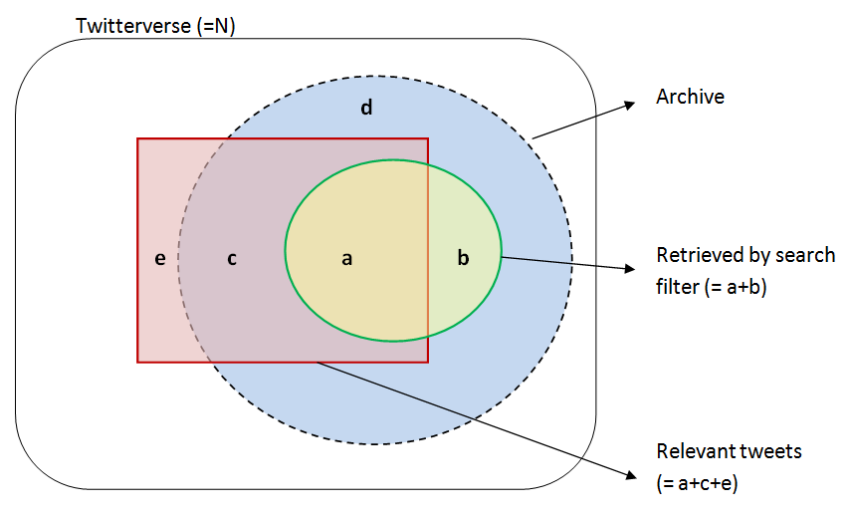
The archive (a+b+c+d), retrieved tweets (a+b), and relevant tweets (a+c+e) in Twitterverse.

Figure 1 displays a structure of data archive, search filter, and relevant tweets in the Twitterverse. The archive contains data returned by broad search terms (the blue circle with dotted line indicates the archive, and the red rectangle indicates all tweets relevant to a specific topic). The search filter returns “a + b” tweets. The archive may omit a small fraction of topic-relevant tweets “e” due to unknown terms, misspellings, etc.

### Assess Search Filter

#### Quality Measures: Definition

Any search filter should be validated based on its ability to distinguish relevant and irrelevant messages. Two criteria are typically used: *retrieval recall* and *retrieval precision* [25]. Precision measures how much of the retrieved data is not garbage. Recall measures how much of the relevant data is retrieved.

Table 2 is constructed to evaluate a search filter against human coding. Precision is a conditional probability that a particular post is relevant, given that it is retrieved, calculated by a/(a + b). Recall is a conditional probability that a particular post is retrieved given that it is relevant, calculated by a/(a + c). Precision is also called positive predictive value, and recall is often called sensitivity of search filter [30]. There is trade-off: high recall may be achieved at the expense of low precision (or low specificity), and vice versa. The F-score is used to report a single measure combining precision and recall [31], computed by:

F=(1 + β^2^)(precision)(recall)/(β^2^ precision + recall) (1)

Often β=1 is used and such measurement is called an F1 score. It can be shown that, using the Bayes’ theorem [32], the recall can be computed by:

Recall=(precision)P(retr)/[(precision)P(retr) + P(relevant|unretr)(1 ‒ P(retr))] (2)

P(retr) denotes the proportion of tweets retrieved, and P(relevant|unretr) denotes the proportion of unretrieved tweets found to be relevant.

Beyond precision and recall, specificity and negative predictive value (NPV) may be used. Specificity measures how much of the irrelevant tweets is discarded, defined by d/(b + d), and is closely related to precision. NPV is the proportion of unretrieved tweets found to be irrelevant, defined by d/(c + d). Note that P(relevant|unretr)=1‒NPV. The proportion of relevant tweets may be obtained by (a + c)/n assuming that the data represent a random sample of the population and human coding is not subject to errors.

**Table 2:** Assessment of search filter with human coding as a gold standard.

Search filter	Human coding		Total
	Coded relevant	Coded not-relevant	
Retrieved	a (True Positive)	b (False Positive)	a + b=n_1_
Not retrieved	c (False Negative)	d (True Negative)	c + d=n_2_
Total	a + c	b + d	n

#### Sampling Plan for Human Coding

Calculation of retrieval precision and recall depends on the assessment of relevant versus irrelevant content. Typically, trained coders inspect a sample of retrieved data to manually evaluate relevancy as well as a sample of unretrieved data. This poses two important questions: how to sample and how many messages to sample. A practical sample size should be determined because it is labor intensive and time consuming to manually code millions of messages, and the estimates of precision and recall should be precise.

**Figure 2:**
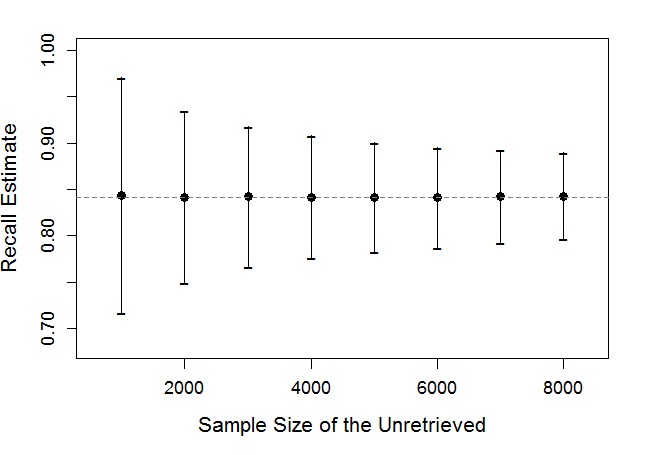
The average limits of 95% confidence intervals for recall (vertical axis) as the sample size of unretrieved messages increases (horizontal axis), fixing the sample size of retrieved data at 3000.

We suggest stratified sampling with retrieval status as strata and oversampling the retrieved messages. This is because typically the size of retrieved messages is small relative to unretrieved messages (n_1_/n_2_<0.1), and oversampling the retrieved messages ensures a desired level of statistical precision. Retrieval recall is more difficult to accurately estimate than retrieval precision because estimating *c* is often similar to finding a needle in a massive haystack of unretrieved messages. Therefore the statistical precision of recall estimate is affected by the sample size. Figure 2 displays how the average length of confidence intervals for retrieval recall estimates decreases as the sample size of unretrieved messages (=k) increases, while the sample size of retrieved message is fixed. The gain in statistical precision diminishes as the number of unretrieved messages increases, and the gain is minimal above a certain sample size. By conducting a simulation or using power analysis tool, a sample size that satisfies the desired level of statistical precision and feasibility can be determined. Multimedia Appendix 1 describes how Figure 2 was generated and discusses more about sample sizes.

#### Estimation of Retrieval Precision and Retrieval Recall

Calculating retrieval precision and recall is straightforward when (1) human coding performs well as a gold standard and (2) Table 2 is complete. We discuss in detail the cases in which one or both conditions are not satisfied and how to address them.

#### Assuming Human Coding Has No Error

##### Ideal Conditions

The definitions of precision and recall are directly used when the two conditions are met. If stratified disproportionate sampling is used, appropriate weights should be applied to calculate recall. Confidence intervals can be estimated based on usual asymptotic methods [33]. If Equation (2) is used to calculate recall, the interval estimate should account for variances of precision and P(relevant|unretr).

##### Unretrieved Messages Could Not Be Archived

Messages matching search filters may be retrieved directly from a data provider so that only the retrieved messages are archived [11,15,21]. Search filter precision can be estimated, but how do we estimate the recall without knowing *c* and *d*? In this case, the unretrieved total n_2_ may be known approximately. Joseph et al used the Bayesian model to estimate recall and specificity when only n_1_ and n_2_ were given [34]. Bayesian models often provide a feasible solution when insufficient information is contained in data to apply usual methods. Since *a* (thus *b*) can be observed in addition to n_1_ and n_2_, we slightly modify their method.

Let *π* be the prevalence of relevant messages, *S* be recall, and *C* be specificity of search filter. The counts of tweets (*a*, *b*, *c*, *d*) in Table 2 have multinomial distribution with respective probabilities forming the likelihood function. Beta prior distributions for *π, S,* and *C* seem sensible because its domain of positive density is bounded in (0,1). Let Beta(α_*π*_, β_*π*_), Beta(α_*S*_, β_*S*_), and Beta(α_*C*_, β_*C*_) denote the prior distributions of *π, S,* and *C* respectively, where Beta(α, β) is beta density function with parameters α and β. Full conditional posterior distributions can be derived for all unknown quantities including *c*, and realized values are sampled from the posterior distributions using a Gibbs sampler. A Gibbs sampler draws from each full conditional posterior distribution sequentially, conditional on all other sampled quantities [32]. It can be shown that the prevalence of relevant messages and recall of search filter have the following posterior distributions: *π* ~ Beta (a + c + α_*π*_, n ‒ a ‒ c *+* β_*π*_), S ~ Beta (a + α_*S*_ , c + β_*S*_).

The quantity *c* is obtained in a previous sampling step. The Bayesian credible interval for an unknown quantity can be obtained based on the random draws from posterior distributions. The Gibbs sampling steps for all unknown quantities are provided in Multimedia Appendix 2.

#### Assuming Human Coding Is Subject to Error

##### Human Coding Is a Silver Standard

Evaluating search filters using imperfect human coding gives a biased impression of data quality. Recall and precision of a search filter depend on recall and specificity of the gold standard [27]. Staquet et al considered a situation where a gold standard has 100% specificity and unknown recall. It may be relatively unlikely that a trained coder evaluates a given irrelevant tweet as relevant. For example, a coder likely will not determine the message “Come get a *smoking* hot jerk chicken wrap from us” as relevant to tobacco smoking. Thus it may be safe to assume, for a given topic, that the specificity of human coding is (close to) 100%. When this assumption is met, the search filter’s recall is unbiased and the bias-corrected equation for precision is given by precision=a/[S_2_ (a + b)], where S_2_ denotes the recall of human coding. Therefore, when human coding does not have perfect recall (false negative error), the method assuming the ideal conditions underestimates search filter precision.

##### Human Coding Is Not a Standard Classifier

Although in many cases human coding serves as a gold/silver standard, it may be an inadequate standard classifier for some topics because human language can be ambiguous (eg, “Leo DiCap is smoking”). Language used on Twitter is often colloquial and creative, and it may be difficult (or impossible) to interpret meaning within 140 characters without looking at related conversations (eg, “I can’t tell if that’s a chocolate Dutch my”; this was a reply to a tweet about Dutch chocolate-flavored cigarillo). Also, coders simply get tired. As a result, coders may falsely determine irrelevant posts to be relevant or vice versa (false positive and false negative error). Joseph et al extended the Bayesian model to the situation where results of two filters, neither of which was a gold standard, were available [34]. We again modify their method to estimate search filter precision and recall.

Similar to Table 2, search filter and human coding results are cross-tabulated. Each cell can be split into truly relevant versus irrelevant contents (see Table 3). Let y_1_ be the count of relevant messages out of the *a* messages retrieved by search filter and human-coded relevant; the count of irrelevant messages is a ‒ y_1_. The rest of the cells can be similarly split.

**Table 3:** Multinomial likelihood contributions of all possible cases of observed data and unknown quantities (the unknown quantities of truly relevant tweets are denoted by y_1_, y_2_, y_3_, y_4_).

Search filter (*j*=1)	Human coding (*j*=2)	
	Coded relevant	Coded not-relevant
Retrieved	a − y_1_	b − y_2_
	y_1_	y_2_
Not retrieved	y_3_	y_4_
	c – y_3_	d − y_4_

Let *π* be the prevalence of relevant messages, S_1_ and C_1_ be recall and specificity of search filter, and S_2_ and C_2_ be recall and specificity of human coding. The eight cells in Table 3 can be expressed as occurrences of multinomial events with probabilities that are functions of the five parameters. Again, a beta distribution can be used to set up prior distribution of each parameter. Denote S_1_, S_2_, C_1_, and C_2_ are distributed Beta(α_S1_, β_S1_), Beta(α_S2_, β_S2_), Beta(α_C1_, β_C1_), and Beta(α_C2_, β_C2_), respectively. It can be shown that the prevalence of relevant messages and search filter recall and specificity have the following posterior distributions:

*π* ~ Beta (∑y_*i*_ + α_*π*_ , n ‒ ∑y_*i*_ + β_*π*_) for *i*=1,2,3,4

S_1_~ Beta (y_1_+ y_2_+ α_S1_, y_3_+ y_4_+ β_S1_)

C_1_~ Beta (c + d ‒ y_3_‒ y_4_+ α_C1_, a + b ‒ y_1_‒ y_2_+ β_C1_)

The precision and NPV of search filter can be obtained by the equations:

Precision_1_=S_1_ *π*/[S_1_ *π* + (1 ‒ C_1_)(1 ‒ *π*)]

NPV_1_=C_1_(1 ‒ *π*)/[C_1_(1 ‒ *π*) + (1 ‒ S_1_) *π*]

These are based on the random draws from posterior distributions of *π*, S_1_, and C_1_. Multimedia Appendix 3 describes the Gibbs sampling steps to obtain random draws from posterior distributions of all unknown quantities including precision and recall of human coding.

## Results

### Develop Search Filter

We obtained Twitter data via an API called Firehose from Gnip, Inc., licensed to provide access to the full stream and historic archive of Twitter data. Access to Firehose is not free as opposed to publicly available data streams such as Streaming API. The Twitter Firehose returned 3,954,575 unique tweets that matched broad keywords about tobacco smoking in October 2012, forming an archive. The archive provided a base to construct Table 2.

We developed a search filter to retrieve e-cigarette-related contents, building around three categories of e-cigarette-related tweets: alternative terms and device parts of e-cigarettes, brand names, and related behavior. We tested keywords using the Twitter Search Engine [35] without logging into our Twitter accounts to avoid search bias. We screened and discarded keywords that returned irrelevant tweets higher than 70% of the time or that returned <10 tweets over a month. When unknown but seemingly relevant terms and phrases that co-occur with our keywords emerged, we checked them in an urban dictionary and other social media platforms, added them to the list, and screened them on Twitter Search. We repeated Steps 1-4 from Table 1 until no more seemingly important keywords were found.

The resulting keyword list included singular and plural forms of e-cigarette terms, different verb forms of behavior terms, and frequent misspellings. We filtered out tweets containing the keywords “atomizer” AND “perfume” as those were likely to describe perfume bottles. Those tweeted by or mentioning @blucigs, an e-cigarette promoting account, were collected. The final list of keywords and rules is presented in Multimedia Appendix 4.

### Assess e-Cigarette Search Filter

#### Sampling Plan for Human Coding

We conducted stratified sampling with retrieval status as strata. A small simulation was performed to determine sample size in each stratum. Data were generated assuming that N was 4 million, retrieval precision was 95%, and retrieval recall was 84%. The simulation details are described in Multimedia Appendix 1 (Case 1). Based on the simulation, we determined that random sampling above 4000 from retrieved tweets and above 6000 from unretrieved tweets would be sufficient.

#### Assuming Human Coding Has No Error

##### Ideal Conditions

The e-cigarette search filter retrieved 82,205 tweets from the archive, yielding P(retr)=0.0208. We randomly sampled 4373 from the retrieved set and coded 4176 of those as relevant, resulting in 95.5% retrieval precision (95% CI 94.9-96.1). Table 4 represents number of tweets cross-tabulated by human coding and search filter; the amount of retrieved tweets was adjusted for the disproportionate sampling fraction. Out of 6305 randomly sampled unretrieved tweets, 20 were found relevant, yielding P(relevant|unretr)=0.0032. The retrieval recall was 86.37% (95% CI 81.4-91.9) by Equation (2). The F1 score was 90.7%.

**Table 4:** Search filter versus human coding on sampled data adjusted for sampling fraction.

Search filter	Human coding		Total
	Coded relevant	Coded not-relevant	
Retrieved	128	6	134
Not retrieved	20	6285	6305
Total	148	6291	6439

##### Unretrieved Messages Could Not Be Archived

To demonstrate the method, we assumed that the archive contained only the tweets retrieved by the e-cigarette search filter. After assigning initial values (Multimedia Appendix 2), a value of precision was sampled from the uniform distribution with limits equal to the 95% confidence interval of the precision (94.9-96.1). We used n_1_=82,205 and n_2_=3,872,370 in the subsequent steps. The Gibbs sampler was repeated 100,000 cycles, and the first 10,000 cycles were discarded as burn-in. The prior distribution and posterior inference results are presented in Table 5. Prevalence indicates the proportion of e-cigarette–relevant tweets within the archive. Prior distributions have been set based on our experience: the specificity is usually high due to low prevalence, and we are confident that the search filter captures the majority of e-cigarette tweets. Although rather high uncertainty was reflected in the prior density of recall—as low as 34%. The F1 score values are computed applying the sampled values of recall and precision on Equation (1) at the end of each cycle. The posterior mean of retrieval recall is 75%: between 50% and 98% with 95% probability. Having no information on the amount of false negative tweets caused a wider interval.

**Table 5:** Prior and posterior means and 95% credible intervals when unretrieved messages cannot be archived.

		Beta prior distribution		Posterior distribution	
		Mean	95% HD^a^	Mean	95% HPD^b^
Prevalence		0.010	1×10^‒6^-0.031	0.028	0.020-0.038
**Search filter**					
	Recall	0.667	0.340-0.954	0.752	0.505-0.979
	Precision^c^	–	–	0.955	0.949-0.961
	Specificity	0.733	0.474-0.962	0.999	0.999-0.999
	F1 score^c^	–	–	0.835	0.663-0.968

^a^HD: highest density interval.

^b^HPD: highest posterior density interval. HPD interval gives narrower length than equal-tailed intervals for skewed distribution (computed using R Package BOA [36]).

^c^Prior density functions of precision and F1 score are not specified but determined as a function of other parameters.

#### Assuming Human Coding Is Subject to Error

##### Human Coding Is a Silver Standard

We assumed that the coders could accurately evaluate irrelevant contents with 100% specificity although they might falsely determine relevant contents to be irrelevant (<100% recall). When human coders make false negative errors, the method assuming the ideal conditions underestimates retrieval precision of search filter. The bias-corrected equation gave the precision of 95.7%, indicating that precision determined assuming the two conditions was minimally biased.

##### Human Coding Is Not a Standard Classifier

Finally we assumed that coders could falsely determine irrelevant contents to be relevant and vice versa (<100% recall and <100% specificity). Each cell of Table 4 can be split into truly relevant and irrelevant tweets. Again let y_1_ be the count of relevant tweets among those retrieved by search filter and human-coded relevant; the count of irrelevant tweets is 128 ‒ y_1_. The Gibbs sampler (see Multimedia Appendix 3) was repeated 100,000 cycles, and the first 10,000 cycles were discarded as burn-in. The prior distribution and posterior inference results are presented in Table 6. Our belief that human coding is slightly better than the search filter is reflected in the prior distributions. The posterior mean of prevalence of e-cigarette tweets is 2% in the archive. The posterior mean of retrieval recall is 93% for the search filter and 96% for human coding. Having more information resulted in smaller uncertainty (ie, shorter HPD intervals).

**Table 6:** Prior and posterior means and 95% credible intervals when human coding is not a standard classifier.

		Beta prior distribution		Posterior distribution	
		Mean	95% HD^a^	Mean	95% HPD^b^
Prevalence		0.019	1×10^‒6^-0.031	0.021	0.018-0.025
**Search filter**					
	Recall	0.667	0.340-0.954	0.929	0.862-0.992
	Precision^c^	–	–	0.956	0.914-0.994
	Specificity	0.733	0.474-0.962	0.999	0.998-1.000
	F1 score^c^	–	–	0.942	0.901-0.982
**Human coding**					
	Recall	0.733	0.474-0.962	0.961	0.923-0.995
	Precision^c^	–	–	0.897	0.824-0.971
	Specificity	0.800	0.616-0.975	0.998	0.996-0.999
	F1 score^c^	–	–	0.927	0.883-0.971

^a^HD: highest density interval.

^b^HPD: highest posterior density interval. HPD interval gives narrower length than equal-tailed intervals for skewed density (computed using R package BOA [36]).

^c^Prior density of precision is not specified but implied as a function of other parameters.

## Discussion

### Principal Findings

While traditional survey data can take years to collect, social media data offer insights into health behavior and public sentiment around health-related topics in a much shorter time frame. They enable researchers to conduct qualitative studies previously only available via focus groups on a large scale. However, a large quantity of data does not assure valid and reliable results. In fact, biases may scale up with the quantity. For example, surveillance systems based on poor data may greatly overpredict or underpredict disease prevalence [37,38]. Without proper search filters, the quality of inferences from social media data will be at best poor, regardless of analytical techniques. Proper filtering and quality assessment are crucial for research with social media data.

Building a search filter is rarely a one-step process, but rather requires significant effort [22]. It is an iterative progression of refining search keywords and rules that capture relevant social data which satisfy pre-specified thresholds for precision and signal to noise ratio. We developed the e-cigarette search filter by monitoring frequency and precision for each keyword. The search filter was refined until no more important new terms were discovered. The keywords were combined with search rules to increase retrieval precision. Wang et al has proposed a method to automatically update the list of keywords by adding the top frequent terms that appear among relevant tweets [28]. We are working toward semi-automating our iterative process by incorporating their method.

We quantified search filter quality by computing retrieval precision and recall in four different cases. Retrieval precision was estimated above 95% in all cases. Retrieval recall was estimated at 86% assuming ideal conditions, 75% when unretrieved messages could not be archived, 86% assuming no false negative errors by coders, and 93% assuming that human coders make both false negative and false positive errors. Researchers should determine which condition is appropriate according to their expert knowledge and experience about the topics and search filters. Regardless of which approach is chosen, the rationale and approach should be clearly reported in any presentation of the data and analyses.

The e-cigarette search filter (see Multimedia Appendix 4) was developed in 2012. Since that time, e-cigarette popularity has increased significantly [39,40], many new brands and various types of vaping devices have entered the market, and e-cigarette-related language and slang terms have evolved. If we were to use the same search filter to study what people say about e-cigarettes on social media in 2015, the retrieval precision and recall would be poor. This underscores the importance of reporting the search filters used, along with their retrieval precision and recall at the time of data collection. When tracking trends of behaviors, attitudes, and beliefs over time, it is crucial to maintain an updated list of keywords/search filters for the given topic.

### Filtering Using Machine Classifiers

Machine learning classifiers are often used for content analysis but also can be used to remove irrelevant messages from the data retrieved by search filters [9,22]. A well-developed classifier can reduce human labor. The accuracy of the classifier should be validated on a hold-out sample by computing precision and recall of the classifier. We refer the validation of classifiers to machine learning literature [31,41,42].

The retrieval precision may be approximated by the classifier precision, but the estimation of retrieval recall can be different from the classifier recall. Classifier recall measures the model’s ability to correctly identify relevant content among the data retrieved by the search filters, whereas retrieval recall estimates how completely relevant content is captured by the search filters, relative to the universe of possible content (all Twitter messages in our example). The estimation of retrieval recall, therefore, is inherently theoretical because it is arduous and resource-intensive to sample unretrieved messages. In practice, its estimation involves examining unretrieved data from as many sources/repositories as possible. Our team collects and manages Twitter data in multiple archives to cover a broad range of topics related to tobacco products and associated behaviors; thus, we could sample from these other archives to see if they captured any content that is potentially relevant to e-cigarettes. Others may archive the Streaming API of Twitter or design another sampling strategy. The important point is to approximate as best as possible the universe in which relevant content may appear.

### Future Research

In addition to data collection and quality assessment, it is important to report data sources, which can affect the validity of inference. Public data on Twitter can be accessed by Firehose, Search API, or Streaming API. The latter two have rate limits, which may prevent retrieval of full data depending on the volume of topics. A small random sample of full stream may contain abundant information about popular topics, for example, a movie star. Some topics may be so scarce in the Twitterverse that rate limit may not be an issue, but sudden spikes in tweet volume induced by, for example, policy change may not be captured due to rate limits. Further research is needed to investigate how the inference is affected by data sources and to provide guidelines. Regardless of data sources, in order to evaluate and compare results across studies, it is critical for researchers using social media data to clearly report how their data were collected and what assumptions were made about unretrieved data, and to provide estimates of the quality of their retrieved data. While strategies may vary by research topic and/or data availability, transparent and thorough reporting is crucial for the credibility of studies as well as the establishment of a rigorous standard for social media research.

###  Limitations

Our methods have certain limitations. We constructed an archive to store tweets potentially related to tobacco smoking. Such an archive is not a random sample of Twitterverse and thus induces selection bias; it may leave out a small fraction of relevant tweets (“e” in Figure 1). This selection bias affects the recall estimate via P(retr) and P(relevant|unretr) in Equation (2). First, if the Twitterverse was used instead of the archive, P(retr) would be much smaller than 0.0208 due to a much larger denominator. This implies that the retrieval recall should be lower. On the other hand, the archive has a high chance of containing e-cigarette messages. That is, it is more likely to contain false negative contents than a random sample of the Twitterverse. Accordingly if the Twitterverse was used, P(relevant|unretr) should be lower and is likely to have many leading zeros. This implies that the retrieval recall should be higher. The two components affect recall estimate in opposite directions. Although the archive has selection bias, it helps find false negative contents and refine the search filter. In addition, the ratio of retrieved to unretrieved messages is relatively larger in the archive than in the Twitterverse. Validating the search filter quality when this ratio is about 1/800 or smaller requires coders to evaluate an impractically huge number of tweets for reliable recall estimation (see Case 2 in Multimedia Appendix 1).

### Call for Rigorous Research

The number of studies that rely on social media data is increasing [43]. However, few have thoroughly described the search filter building process or fully assessed data quality. In order to assess data collection and quality, research involving social media data should clearly describe data sources, including how data were accessed and collected and how search filters were built, as well as presenting retrieval precision and recall. Data with low recall will poorly represent the target topic, and data with low precision will give misleading information. In light of moving toward a reporting standard, we propose a checklist (see Textbox 1) for reporting social media data preparation. Study findings should be replicable and comparable with clearly described data and methods.

**Checklist for social media data preparation and reporting.**

1. Data source

- Social networking site and time frame
- How the data are accessed (eg, Streaming API)
- Why the data source is suitable for the research topics? Is there any limitation with the data source?

2. Development of search filter

- How candidate keywords are generated
- How keywords are refined
- Complete list of final keywords and search rules

3. Assessment of search filter

- Assumptions about human coding
- Sampling frame and sample size for human coding
- Whether all necessary data are available to assess the search filter
- Whether and how retrieval precision and recall are estimated

### Conclusions

In this paper, we proposed a framework for social media data collection and validation and discussed how to quantify data quality under different conditions. Our proposed methodology is not limited to Twitter and can be adapted to other public social networking sites (as opposed to online forums or closed online networks). The length limit of posts, different data fields (title, description, tag, comment, etc), main user characteristics, data streaming, or crawling tools may be considered for modification. Our method is primarily useful for text-based social data, but it can be adapted to image-based social media. Instagram users, for instance, post photos with hashtags; we can retrieve potentially relevant contents based on hashtags [44] and remove irrelevant contents by using an image classifier. We hope our proposed framework and methods contribute to more rigorous and transparent health research using social media data.

## Appendix

Multimedia Appendix 1 The sample size simulation for human coding.

Multimedia Appendix 2 Precision and recall estimation using only retrieved data.

Multimedia Appendix 3 Precision and recall estimation when human coding is not a standard classifier.

Multimedia Appendix 4 E-cigarette search keywords and rules.

## Abbreviations

API: application program interface
FDA: Food and Drug Administration
HD: highest density
HPD: highest posterior density
NIH: National Institutes of Health
NPV: negative predictive value
