# Supplementary material for: Garbage in, Garbage Out: Data Collection, Quality Assessment and Reporting Standards for Social Media Data Use in Health Research, Infodemiology and Digital Disease Detection
Source: J Med Internet Res. 2016 Feb 26;18(2):e41. doi: 10.2196/jmir.4738 (PMC4788740; doi:10.2196/jmir.4738)
Supplement: Multimedia Appendix 1 [file jmir_v18i2e41_app1.pdf]

Multimedia Appendix 1. **The sample size simulation for human coding**

Case 1: We conducted simulation in order to determine sample size for each stratum. Data were generated assuming the population size of 4 million, retrieval precision of 95%, and retrieval recall of 84%. The retrieved (n_1) to the unretrieved (n_2) ratio was 1 to 39, and prevalence was set 2.8%. Let m be the sample size for retrieved data and k be the sample size for unretrieved data. We randomly sampled m out of n_1 and k out of n_2, computed precision and recall on the sampled data, and checked whether their 95% confidence intervals included the true values. We replicated this many times to obtain the average confidence intervals.

The variability of retrieval recall estimate is affected by the size k. Therefore, we repeated the simulation by increasing k at a fixed value of m. Figure 2 displays how the average confidence intervals for recall estimates change as k increases from 1,000 to 8,000 while m is fixed at 3,000. The gain in variability reduction for recall estimate is small when k is above 6000. The simulation results for m=2,000 are presented in the table below; they show a similar pattern. The coverage probability of all precision estimates was satisfactory.

Case 2: We also considered another scenario that the population size was 10 million, retrieval precision and recall were 92% and 85% respectively. The n_1 to n_2 ratio was approximately 1 to 815, and prevalence was set 0.1%. We considered m=600 and k=8,000 to 30,000 because of the large n_2. The coverage probability for interval estimates of recall does not reach the desired level 95% in many cases, the variability around the estimate is still high even when k=30,000.

The simulation suggests that the ratio of n_1 to n_2 affects accuracy of the recall estimates; when the ratio is tiny, taking a sizeable sample of the unretrieved tweets may compensate for inaccuracy. But human coders can code only so much before fatigue interferes. To determine an appropriate sample size for human coding, a balance between the desired level of statistical precision and feasibility should be considered.

Case 1: population size of 4 million, the retrieved to the unretrieved ratio = 1:39, prevalence = .028, precision= .95, recall=.84

Case 2: population size of 10 million, the retrieved to the unretrieved ratio = 1:815, prevalence = .001, precision= .92, recall=.85

Retrieved		Unretrieved		Precision				Recall			
m	%	*k*	%	Mean	95% L	95% U	C(%)	Mean	95% L	95% U	C(%)
**Case 1**											
3000	3	1,000	0.03	0.9500	0.9422	0.9578	95.3	0.8433	0.7163	0.9702	93.8
		2,000	0.05	0.9501	0.9423	0.9579	95.0	0.8418	0.7494	0.9341	95.4
		3,000	0.08	0.9499	0.9421	0.9577	93.6	0.8419	0.7660	0.9177	95.6
		4,000	0.10	0.9501	0.9423	0.9579	94.2	0.8418	0.7759	0.9077	94.8
		5,000	0.13	0.9500	0.9422	0.9578	95.8	0.8412	0.7821	0.9003	96.0
		6,000	0.15	0.9501	0.9423	0.9579	95.1	0.8409	0.7869	0.8950	95.4
		7,000	0.18	0.9502	0.9424	0.9579	95.9	0.8422	0.7922	0.8922	96.8
		8,000	0.21	0.9500	0.9422	0.9578	94.1	0.8427	0.7960	0.8895	96.4
2000	2	1,000	0.03	0.9500	0.9405	0.9595	95.0	0.8474	0.7215	0.9734	93.7
		2,000	0.05	0.9499	0.9404	0.9595	95.2	0.8432	0.7512	0.9352	94.5
		3,000	0.08	0.9498	0.9403	0.9594	97.0	0.8421	0.7663	0.9179	95.8
		4,000	0.10	0.9501	0.9406	0.9597	94.3	0.8414	0.7754	0.9073	94.9
		5,000	0.13	0.9502	0.9407	0.9597	94.7	0.8425	0.7835	0.9015	95.6
		6,000	0.15	0.9499	0.9403	0.9594	93.0	0.8415	0.7875	0.8955	95.5
		7,000	0.18	0.9497	0.9401	0.9592	94.9	0.8410	0.7910	0.8911	95.7
		8,000	0.21	0.9499	0.9404	0.9595	94.7	0.8410	0.7941	0.8879	97.1
**Case 2**											
600	4.9	8,000	0.08	0.9203	0.8987	0.941	94.7	0.8604	0.7306	1.0000	79.8
		10,000	0.10	0.9200	0.8984	0.9417	95.2	0.8572	0.7291	1.0000	87.4
		12,000	0.12	0.9201	0.8985	0.9417	94.7	0.8562	0.7327	1.0000	91.3
		14,000	0.14	0.9203	0.8987	0.9419	95.2	0.8559	0.7371	1.0000	94.0
		16,000	0.16	0.9204	0.8988	0.9420	95.4	0.8540	0.7394	1.0000	82.8
		18,000	0.18	0.9201	0.8985	0.9418	95.4	0.8537	0.7431	0.9994	87.6
		20,000	0.20	0.9201	0.8985	0.9417	94.9	0.8531	0.7462	0.9916	90.1
		22,000	0.22	0.9199	0.8982	0.9416	95.2	0.8528	0.7494	0.9847	93.4
		24,000	0.24	0.9198	0.8981	0.9414	95.4	0.8523	0.7517	0.9788	94.5
		26,000	0.26	0.9201	0.8985	0.9418	95.2	0.8529	0.7554	0.9740	89.7
		28,000	0.28	0.9198	0.8981	0.9415	94.5	0.8530	0.7582	0.9697	91.7
		30,000	0.30	0.9198	0.8981	0.9415	95.0	0.8527	0.7605	0.9652	93.4

m= the sample size of retrieved data, *k*= the sample size of unretrieved data, % = sampling fraction in percentage. Each scenario was repeated 3000 times. The mean of point estimates and mean of 95% confidence limits are reported. The coverage probability C(%) shows how many times the 95% confidence intervals contain the true value.
