# Supplementary material for: Garbage in, Garbage Out: Data Collection, Quality Assessment and Reporting Standards for Social Media Data Use in Health Research, Infodemiology and Digital Disease Detection
Source: J Med Internet Res. 2016 Feb 26;18(2):e41. doi: 10.2196/jmir.4738 (PMC4788740; doi:10.2196/jmir.4738)
Supplement: Multimedia Appendix 2 [file jmir_v18i2e41_app2.pdf]

Multimedia Appendix 2. **Precision and recall estimation using only retrieved data**

First the precision and its 95% confidence interval, denoted by (L_p, U_p), within the retrieved data are calculated. Then our modified method takes the following steps:

(1) Assign initial values of $\pi, S,$ and C.

(2) Sample a value of precision from Uniform (L_p, U_p)

(3) Sample a from Binomial $(n_1, \text{precision})$

Sample c from Binomial $\left(n_2, \frac{\pi(1-S)}{\pi(1-S)+(1-\pi)C}\right)$

(4) Sample π from Beta $(a + c + \alpha_\pi, n - a - c + \beta_\pi)$

Sample S from Beta $(a + \alpha_S, c + \beta_S)$

Sample C from Beta $(n_2 - c + \alpha_C, n_1 - a + \beta_C)$

A value of precision is sampled in step (2) from the Uniform distribution with lower and upper bounds equal to 95% confidence limits of the precision estimate. The steps (2)-(4) are repeated many times. The sampled values from the previous steps are used in the subsequent steps. A few different prior distributions may be tried to achieve robust results. We refer to Joseph et al. (1995) for derivation of posterior distributions [34].
