# Supplementary material for: Garbage in, Garbage Out: Data Collection, Quality Assessment and Reporting Standards for Social Media Data Use in Health Research, Infodemiology and Digital Disease Detection
Source: J Med Internet Res. 2016 Feb 26;18(2):e41. doi: 10.2196/jmir.4738 (PMC4788740; doi:10.2196/jmir.4738)
Supplement: Multimedia Appendix 3 [file jmir_v18i2e41_app3.pdf]

Multimedia Appendix 3. Precision and recall estimation when human coding is not a standard classifier

Our modified method takes the following steps of Gibbs sampling:

(1) Assign initial values for $(\pi, S_1, S_2, C_1, C_2)$.

(2) Sample y_1 from Binomial $\left(a\ ,\ \frac{\pi S_1 S_2}{\pi S_1 S_2+(1-\pi)(1-C_1)(1-C_2)}\right)$.

Sample y_2 from Binomial $\left(b\ ,\ \frac{\pi S_1(1-S_2)}{\pi S_1(1-S_2)+(1-\pi)(1-C_1)C_2}\right)$.

Sample y_3 from Binomial $\left(c\ ,\ \frac{\pi(1-S_1)S_2}{\pi(1-S_1)S_2+(1-\pi)C_1(1-C_2)}\right)$.

Sample y_4 from Binomial $\left(d\ ,\ \frac{\pi(1-S_1)(1-S_2)}{\pi(1-S_1)(1-S_2)+(1-\pi)C_1 C_2}\right)$.

(3) Sample π from Beta $(\sum_i y_i + \alpha_\pi\ ,\ n - \sum_i y_i + \beta_\pi)$ for $i = 1, 2, 3, 4$

(4) Sample S_1 from Beta $(y_1 + y_2 + \alpha_{S1}\ ,\ y_3 + y_4 + \beta_{S1})$

Sample C_1 from Beta $(c + d - (y_3 + y_4) + \alpha_{C1}\ ,\ a + b - (y_1 + y_2) + \beta_{C1})$

Sample S_2 from Beta $(y_1 + y_3 + \alpha_{S2}\ ,\ y_2 + y_4 + \beta_{S2})$

Sample C_2 from Beta $(b + d - (y_2 + y_4) + \alpha_{C2}\ ,\ a + c - (y_1 + y_3) + \beta_{C2})$

(5) Compute precisions by $Precision_j = \frac{S_j \pi}{S_j \pi + (1-C_j)(1-\pi)}$ for $j = 1, 2$

The steps (2)-(5) are repeated a large number of times. The step (5) is due to Bayes' Theorem.

The NPV may be obtained in Step (5) using the equation,

$$NPV_j = \frac{C_j(1-\pi)}{C_j(1-\pi) + (1-S_j)\pi} \quad \text{for } j = 1,2\,.$$

A few different prior distributions and initial values may be tried to achieve robust results.
