# Supplementary material for: Garbage in, Garbage Out: Data Collection, Quality Assessment and Reporting Standards for Social Media Data Use in Health Research, Infodemiology and Digital Disease Detection
Source: J Med Internet Res. 2016 Feb 26;18(2):e41. doi: 10.2196/jmir.4738 (PMC4788740; doi:10.2196/jmir.4738)
Supplement: Multimedia Appendix 4 [file jmir_v18i2e41_app4.pdf]

Multimedia Appendix 4. E-cigarette search keywords and rules

The keywords used to construct the tobacco smoking archive:
smok, smoking, tobbacco, tobbaco, bigtobacco, menthol, cig(s), cigarette(s), cigaret, ciggarete, ciggarette, ciggs, ecig(s), hookah(s), cigar(s), cigarillo(s), antimoking, quit smoking, stop smoking, quitnow, quitline, sgreport, etc.

The e-cigarette search filter:

**Category**	**Search Keywords and Rules**
Alternative terms of e-cigarettes	ecig(s), "e cig(s)", e-cig(s), ecigarette(s), e-cigarette(s), ehookah, e-hookah, ejuice(s), e-juice(s), eliquid(s), e-liquid(s), e-smoke(s), esmoke(s), lavatube(s), smokestik(s)
E-cigarette device parts	cartomizer(s), atomizer(s) if NOT perfume
Specific brand of e-cigarettes	@blucigs, from:blucigs[1], blu cig, blu cigarette, njoy cig, njoy cigarette, "green smoke" "south beach smoke", eversmoke, "Joye 510", joye510, joyetech, logicecig, logicecigs, smartsmoker, "v2 cig(s)", v2cig(s), zerocig(s)
Behavior	vaper(s), vaping

[1] retrieved all tweets posted by @blucigs

The coders were well-trained by working on another study that investigated the electronic cigarettes-related messages on Twitter in 2012 [23]. The coders discussed ambiguous tweets together to reach consensus.
